# Supplementary figures and images for: EPB41L4A-AS1 long noncoding RNA acts in both cis- and trans-acting transcriptional regulation and controls nucleolar biology
Source: eLife. 2026 Mar 10;14:RP106846. doi: 10.7554/eLife.106846 (PMC12975130; doi:10.7554/eLife.106846)

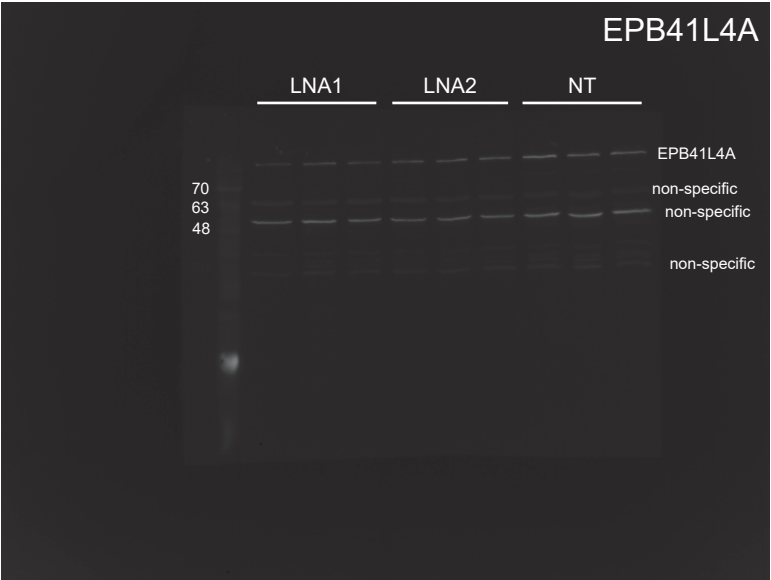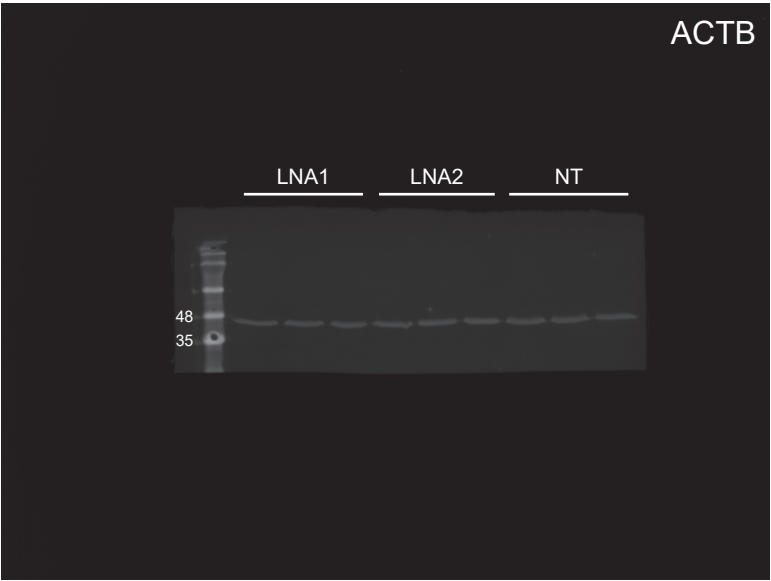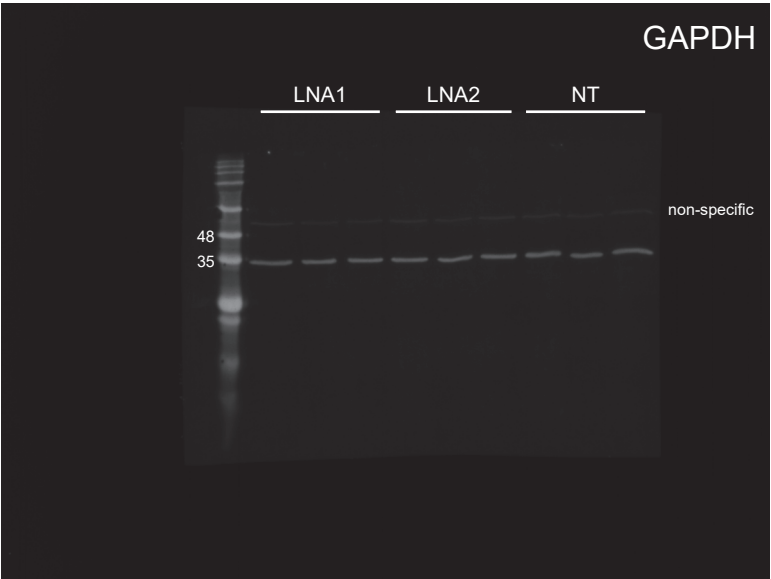

Supplement: Figure 2—source data 1. [file elife-106846-fig2-data1.zip › Figure 2-source data 1.pdf]

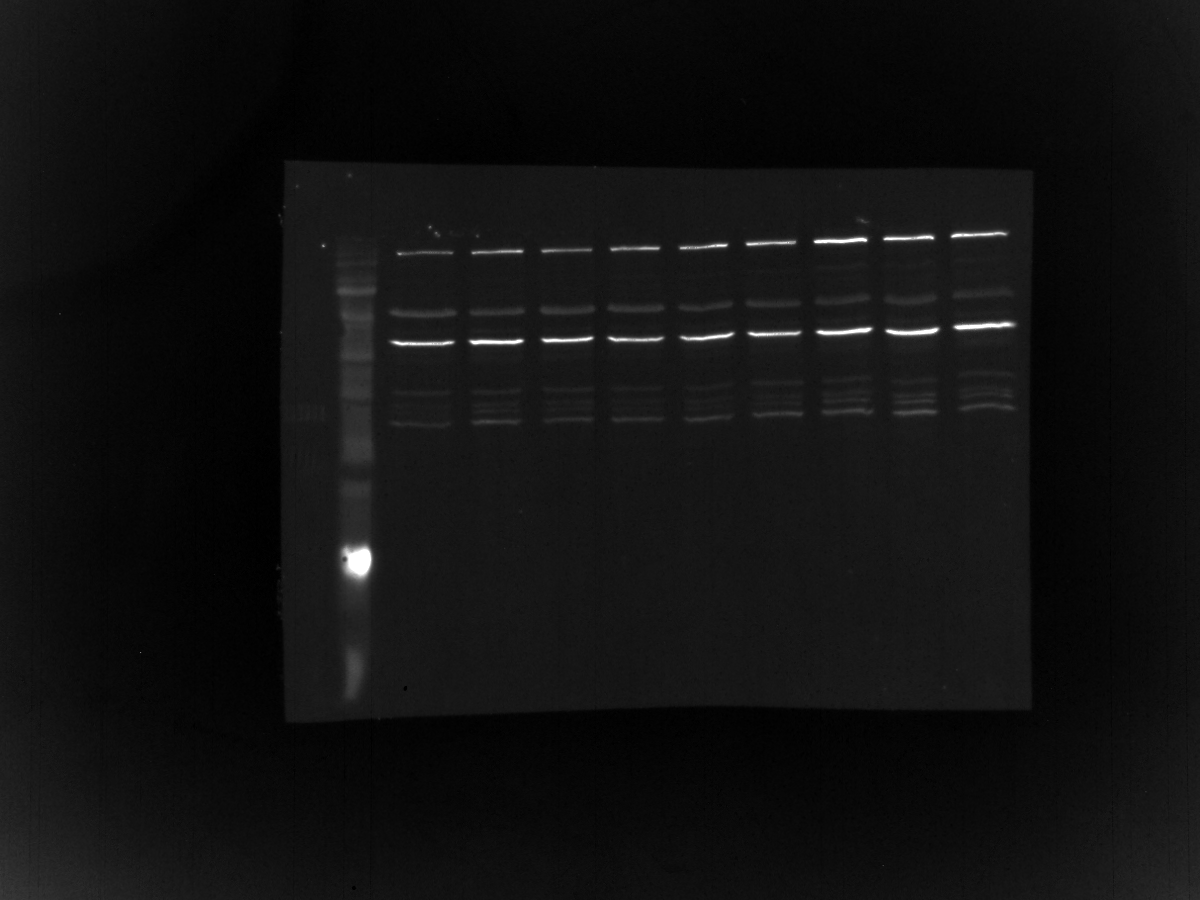

Supplement: Figure 2—source data 2. [file elife-106846-fig2-data2.zip › 2023-0704-130339 (EPB41L4A).tif]

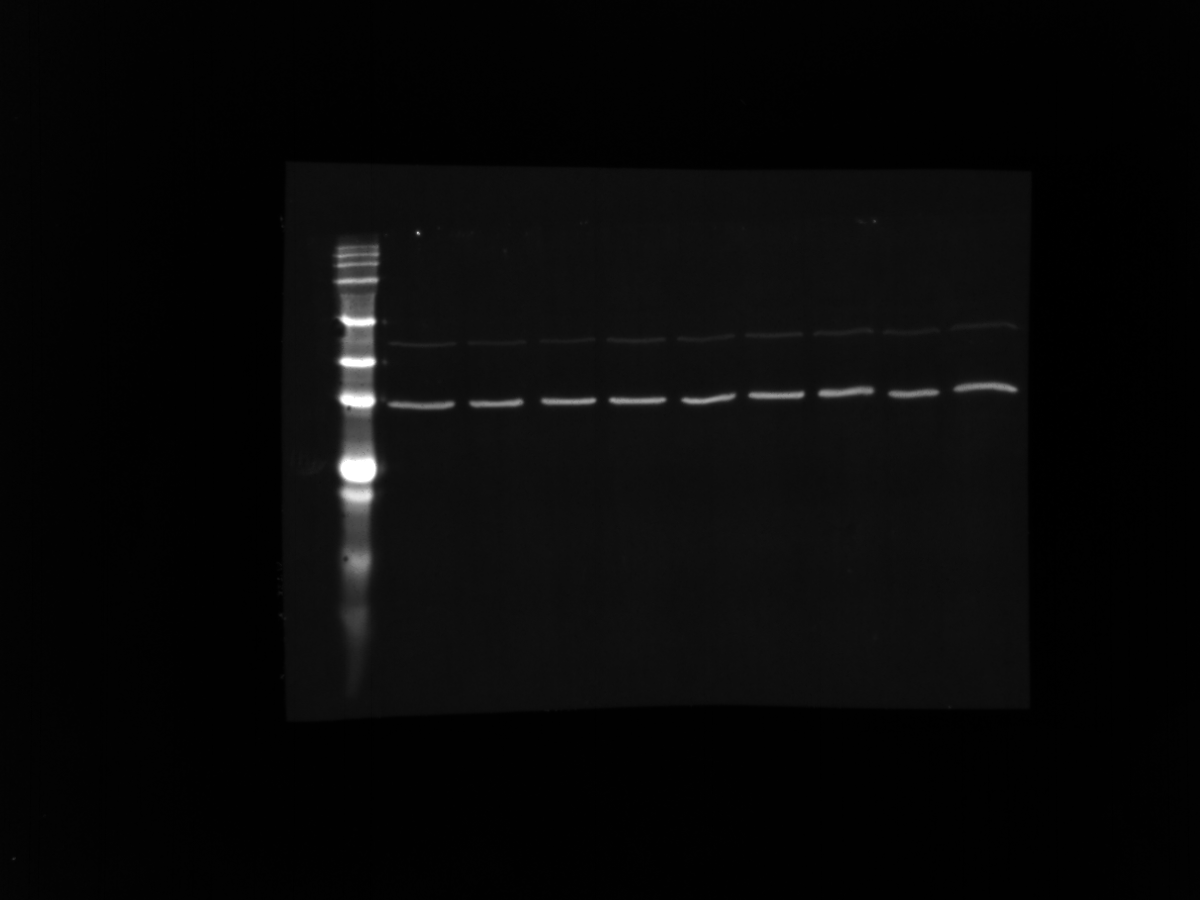

Supplement: Figure 2—source data 2. [file elife-106846-fig2-data2.zip › 2023-0704-130340 (GAPDH).tif]

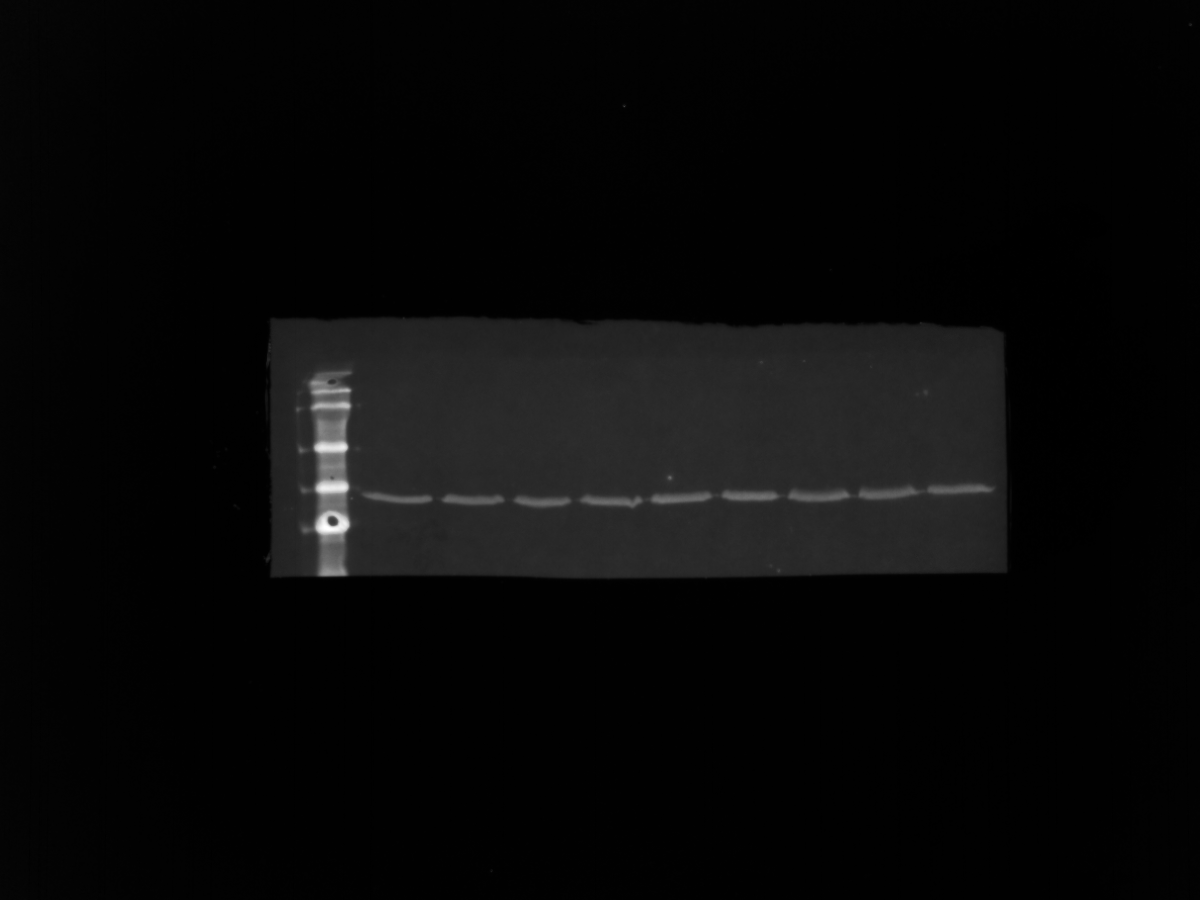

Supplement: Figure 2—source data 2. [file elife-106846-fig2-data2.zip › 2024-0318-122545 (ACTB).tif]

CDKN1A

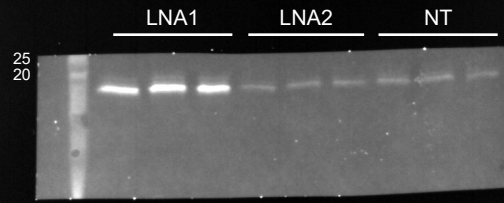

ACTB

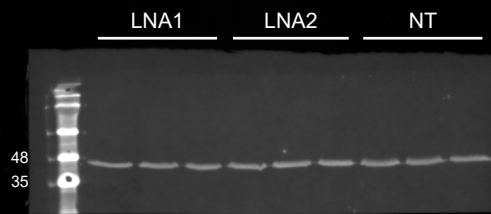

GAPDH

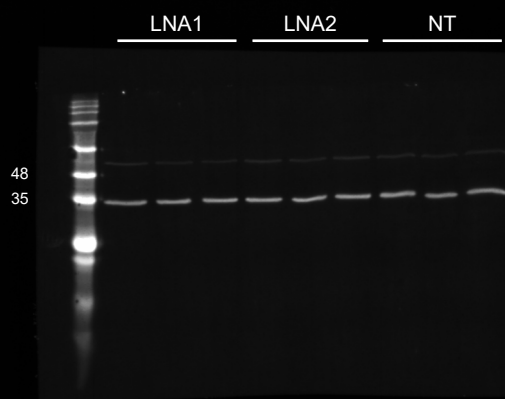

Supplement: Figure 2—figure supplement 2—source data 1. [file elife-106846-fig2-figsupp2-data1.zip › Figure 2-figure supplement 2-source data 1.pdf]

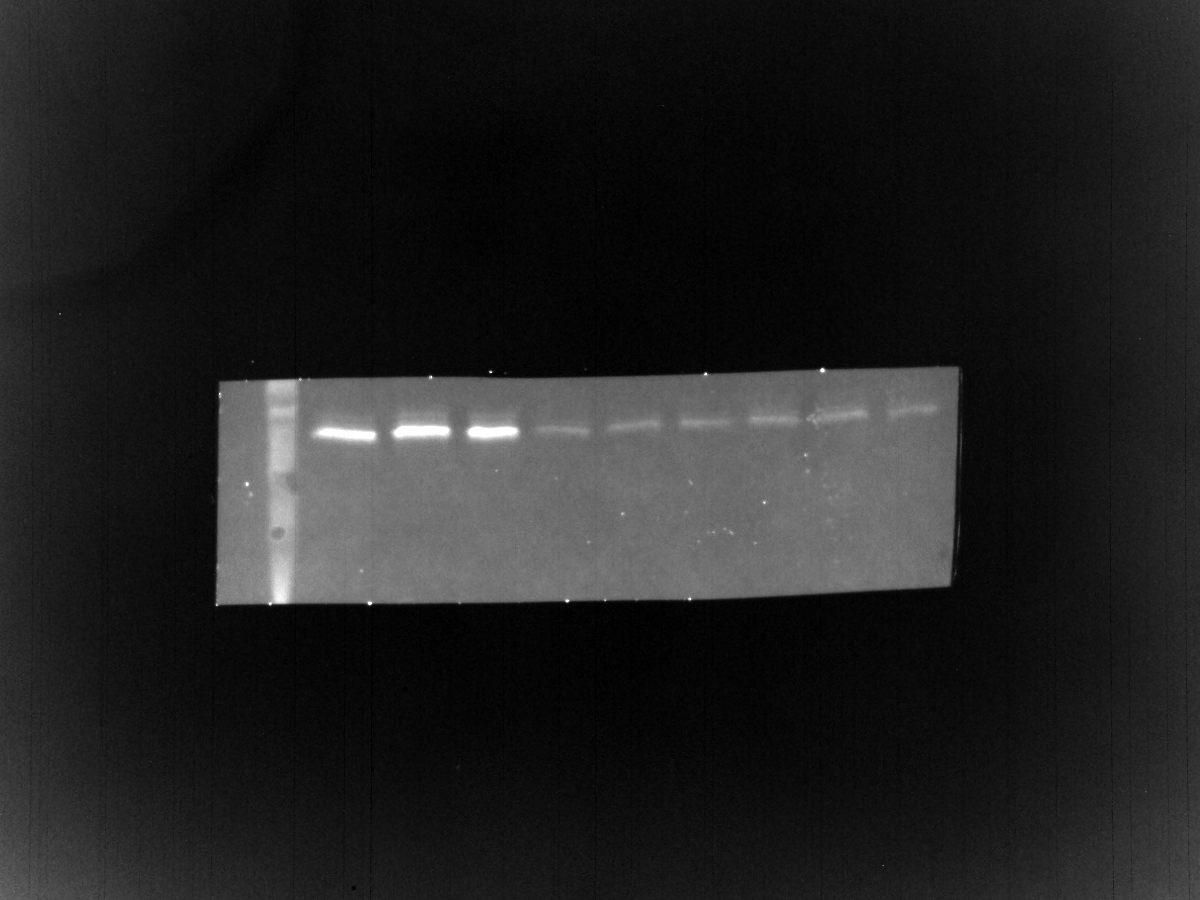

Supplement: Figure 2—figure supplement 2—source data 2. [file elife-106846-fig2-figsupp2-data2.zip › 2024-0318-120214 (CDKN1A).tif]

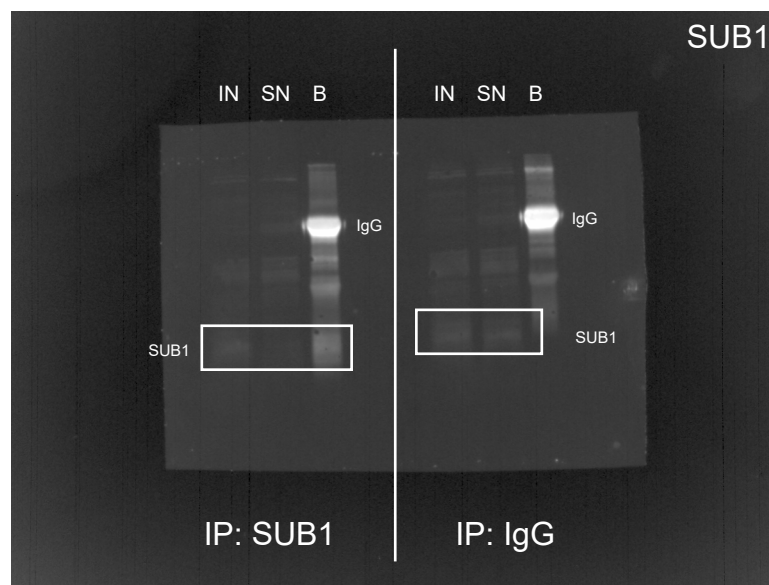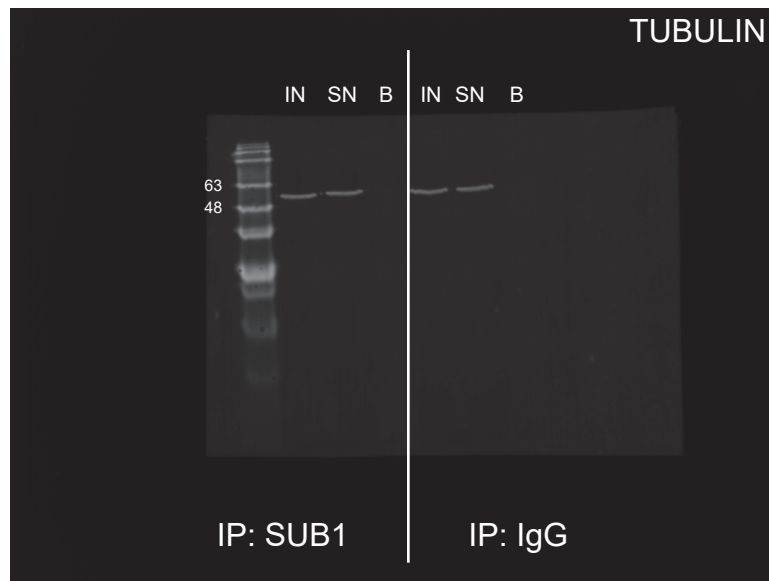

IN: input; SN: supernatant, B: bound

Supplement: Figure 3—source data 1. [file elife-106846-fig3-data1.zip › Figure 3-source data 1.pdf]

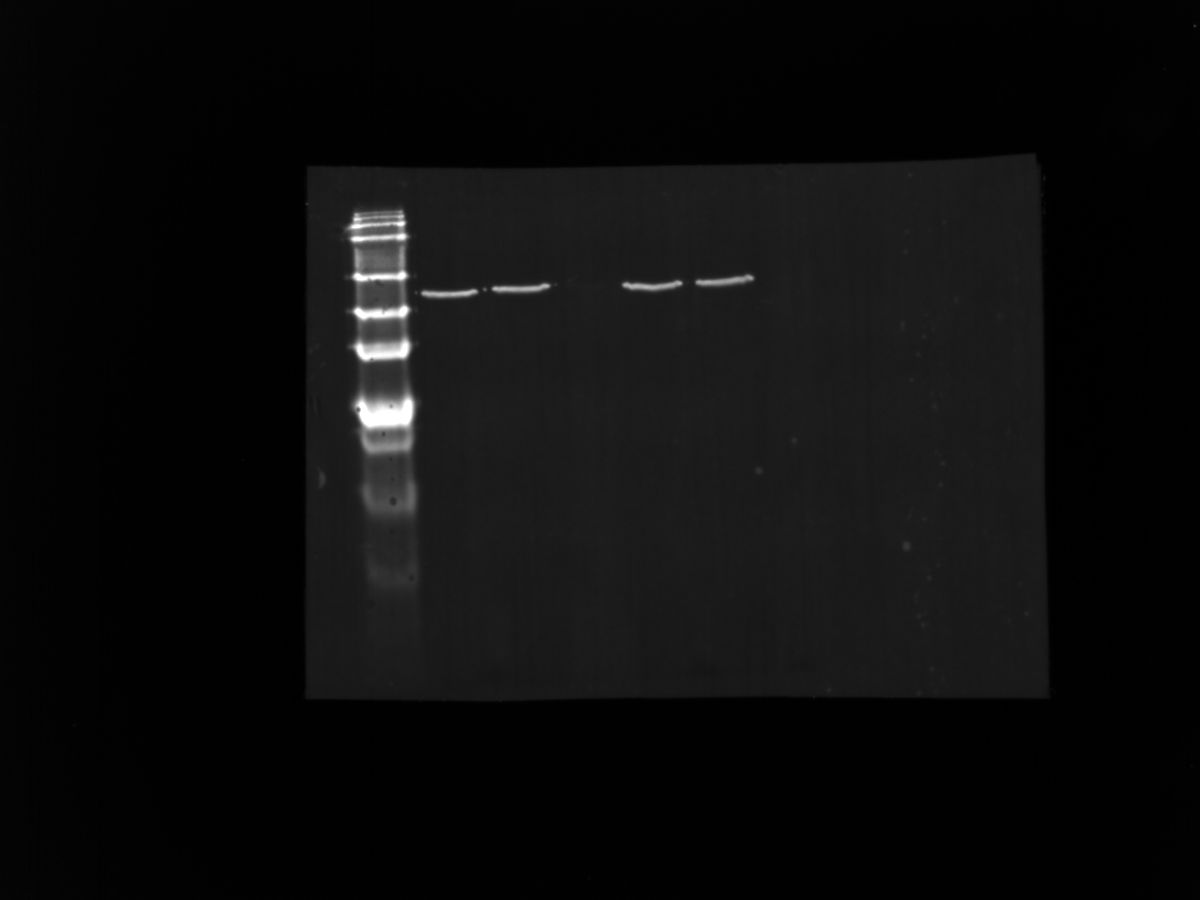

Supplement: Figure 3—source data 2. [file elife-106846-fig3-data2.zip › 2022-0706-102018 (TUBULIN).tif]

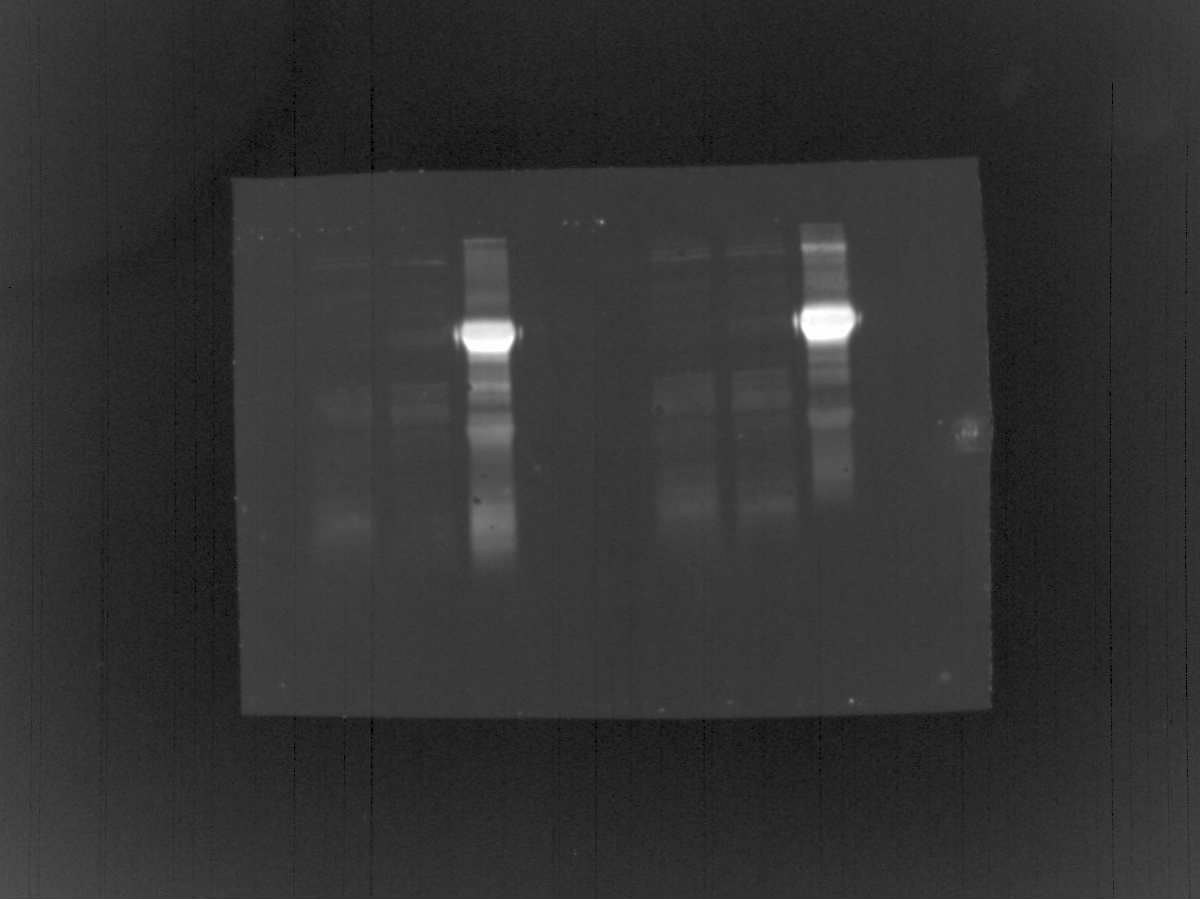

Supplement: Figure 3—source data 2. [file elife-106846-fig3-data2.zip › 2022-0713-103038 (SUB1).tif]

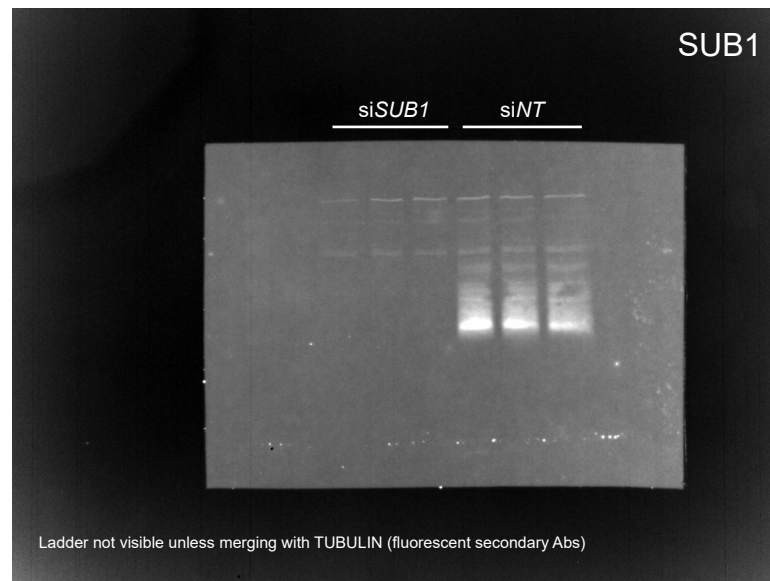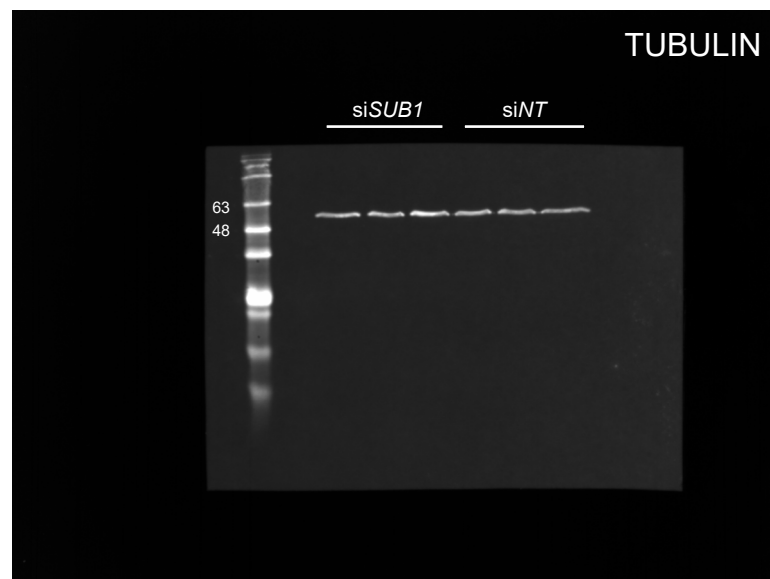

Supplement: Figure 4—figure supplement 1—source data 1. [file elife-106846-fig4-figsupp1-data1.zip › Figure 4-figure supplement 1-source data 1.pdf]

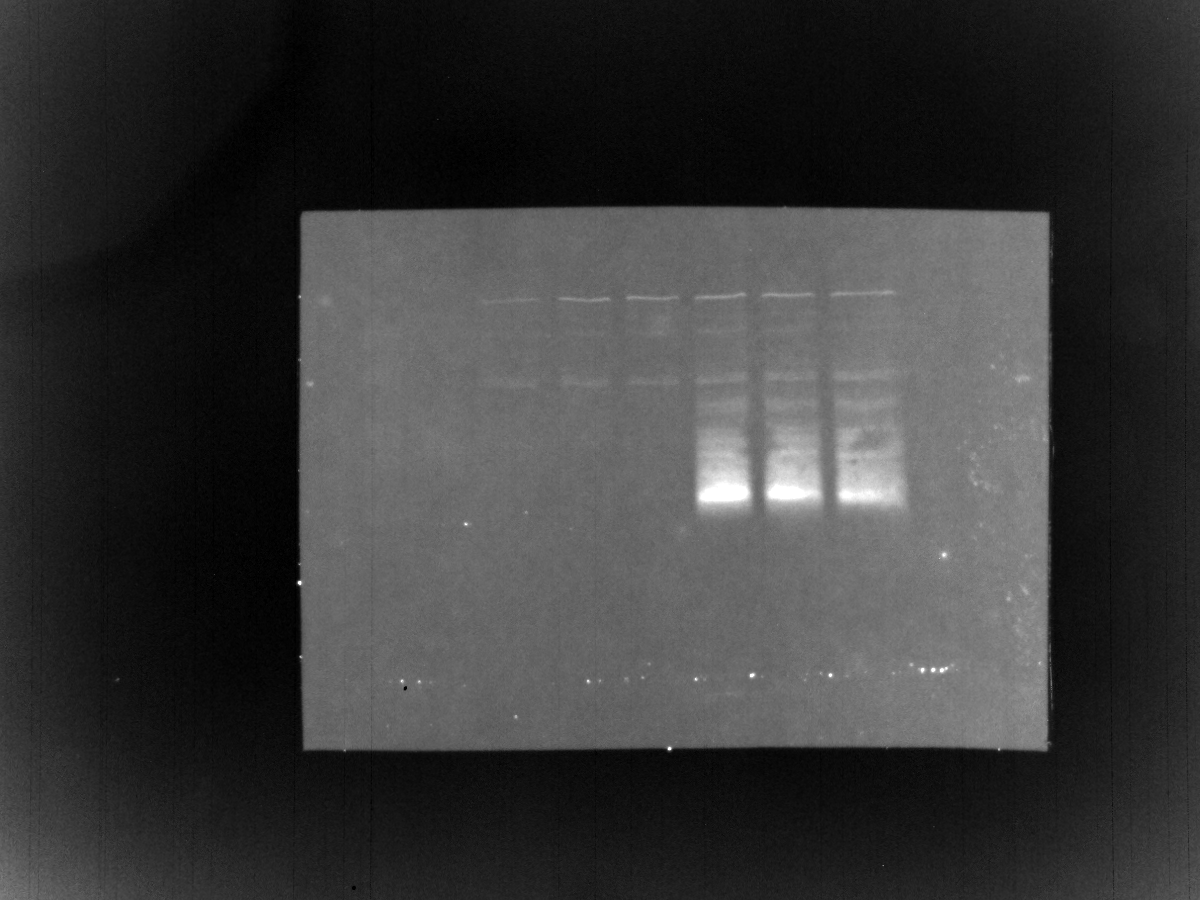

Supplement: Figure 4—figure supplement 1—source data 2. [file elife-106846-fig4-figsupp1-data2.zip › 2023-0503-100656 (SUB1).tif]

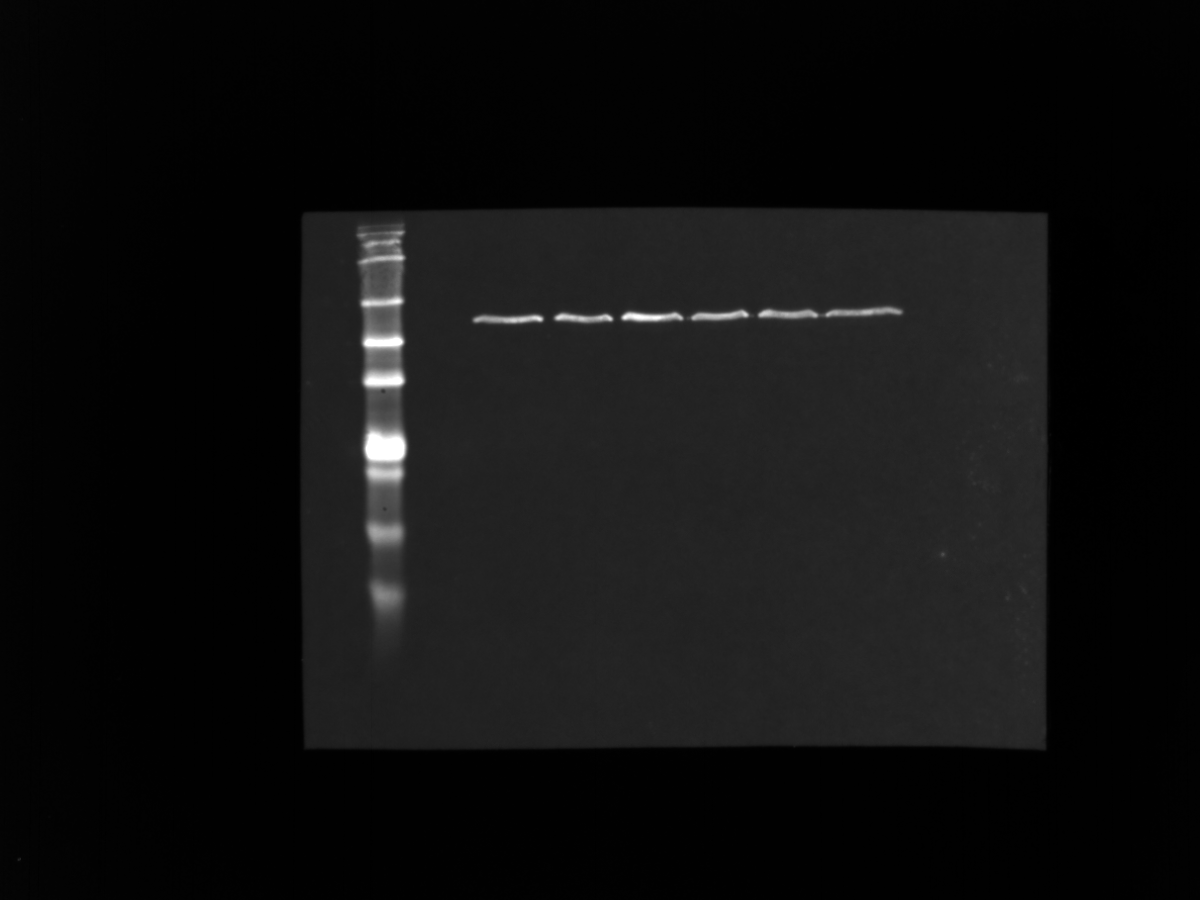

Supplement: Figure 4—figure supplement 1—source data 2. [file elife-106846-fig4-figsupp1-data2.zip › 2023-0503-100657 (TUBULIN).tif]

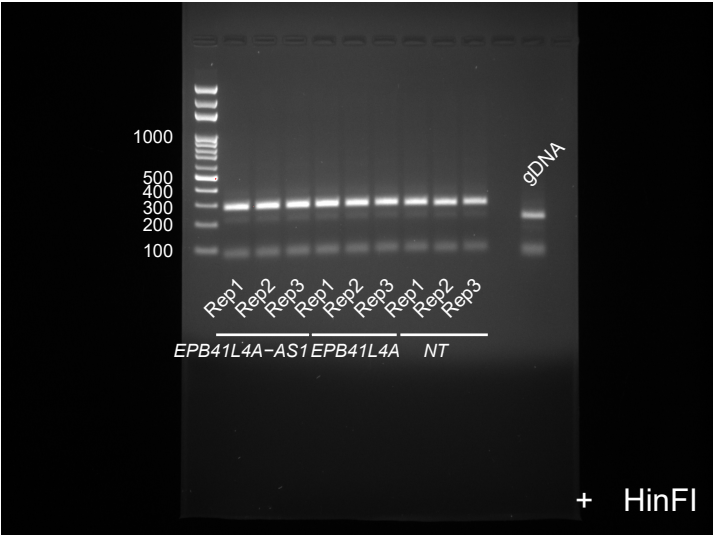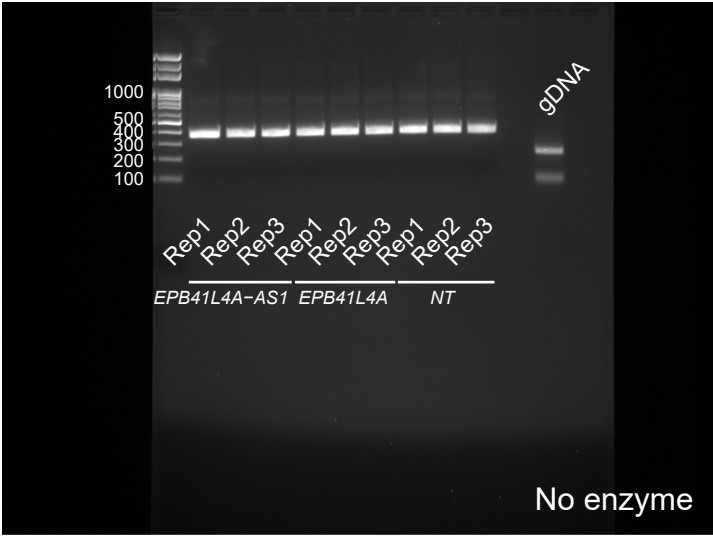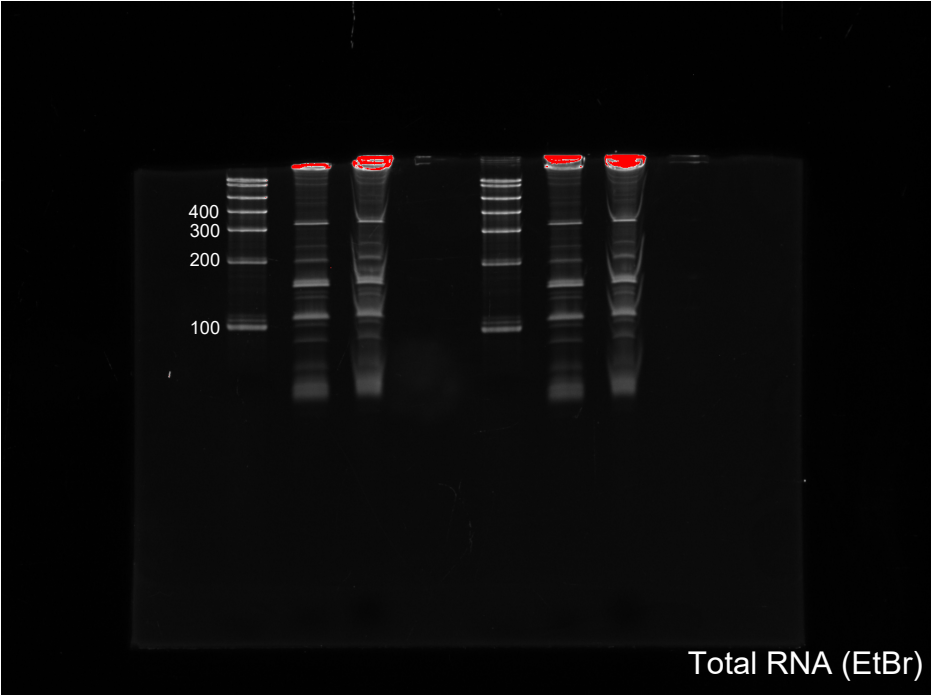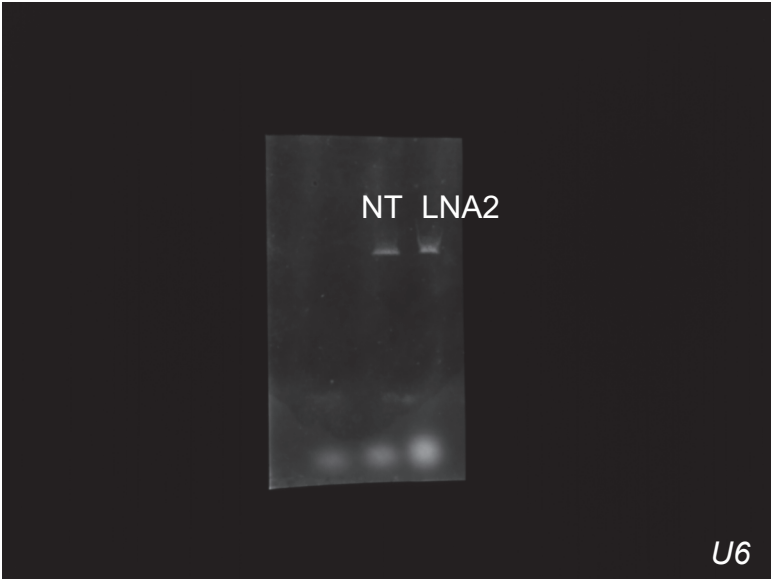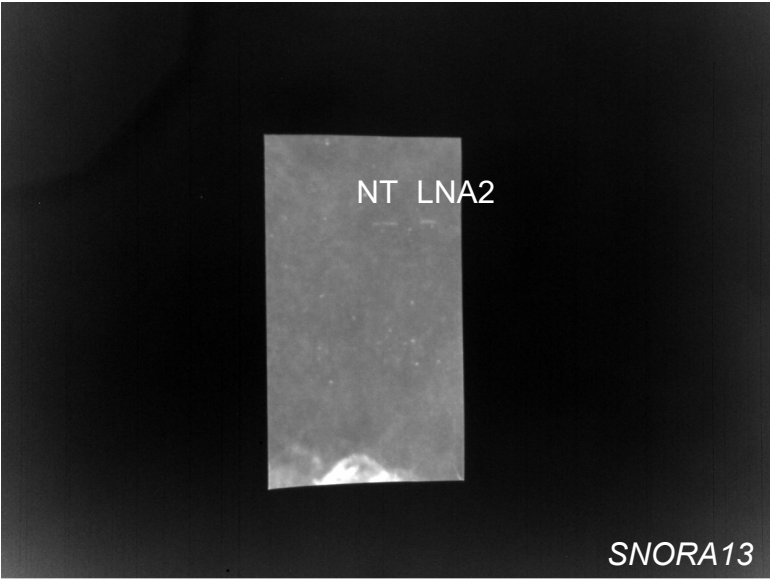

Supplement: Figure 6—source data 1. [file elife-106846-fig6-data1.zip › Figure 6-source data 1.pdf]

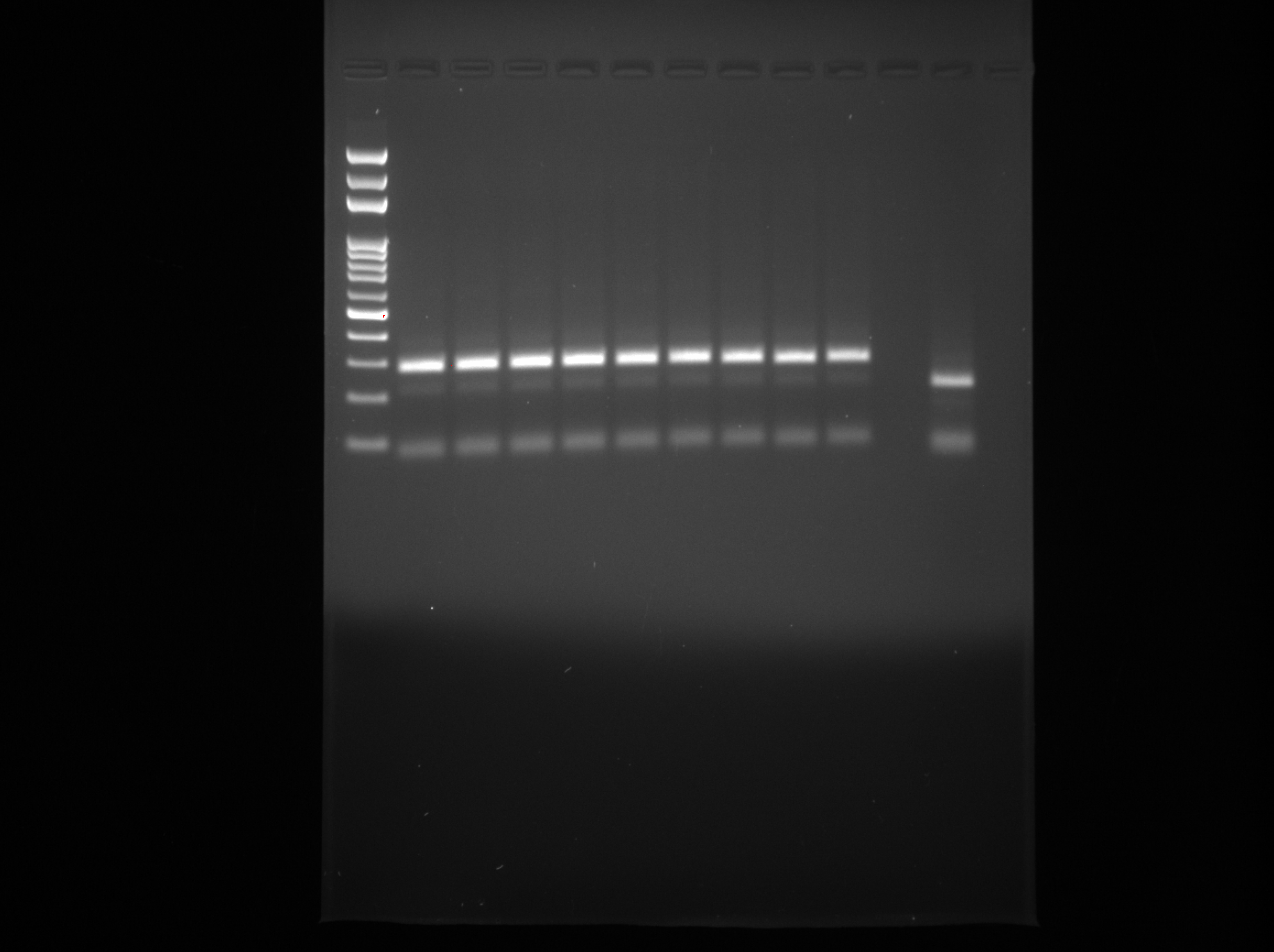

Supplement: Figure 6—source data 2. [file elife-106846-fig6-data2.zip › 18S MACP digestion.tif]

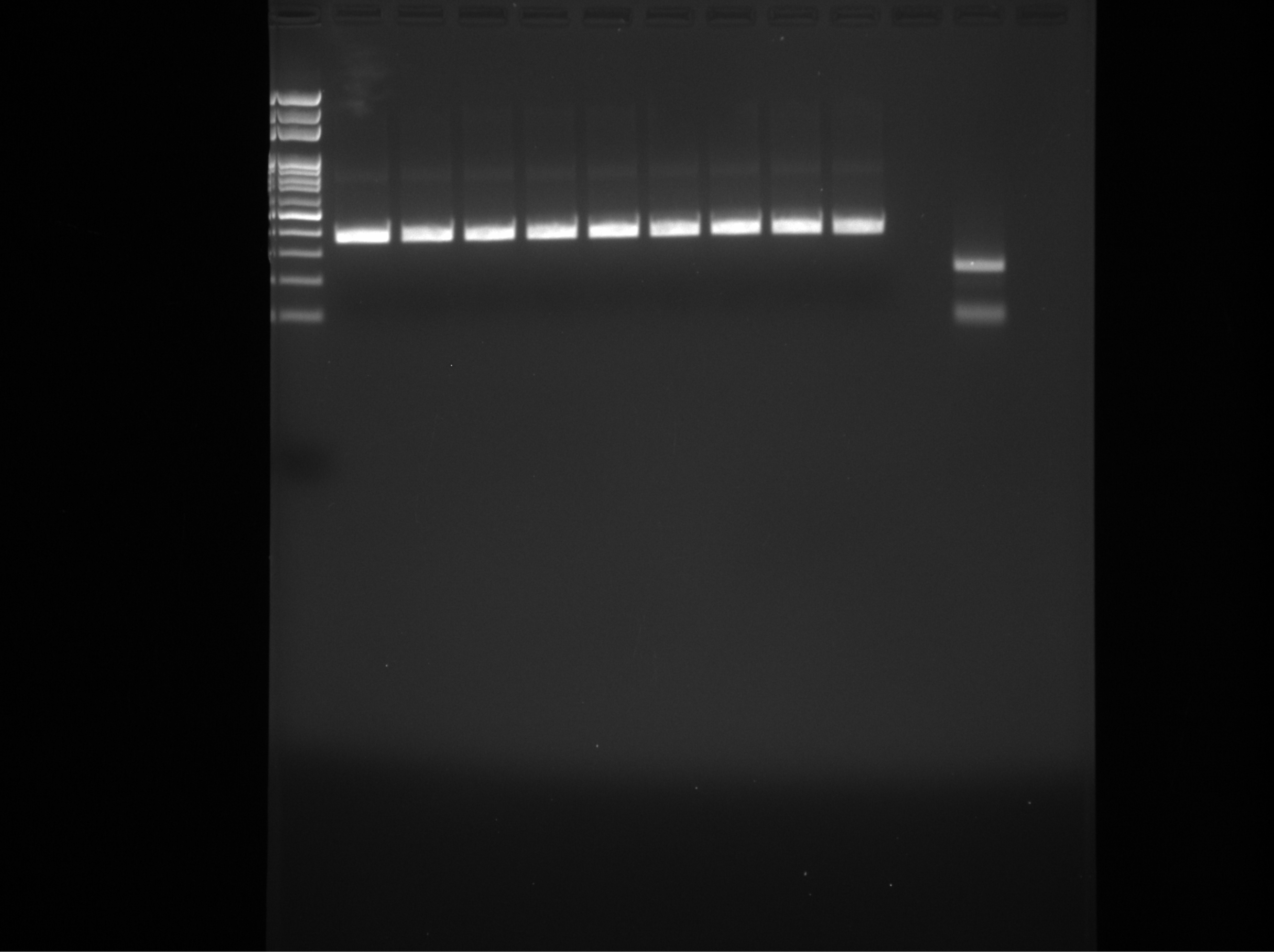

Supplement: Figure 6—source data 2. [file elife-106846-fig6-data2.zip › 18S MACP no enzyme.tif]

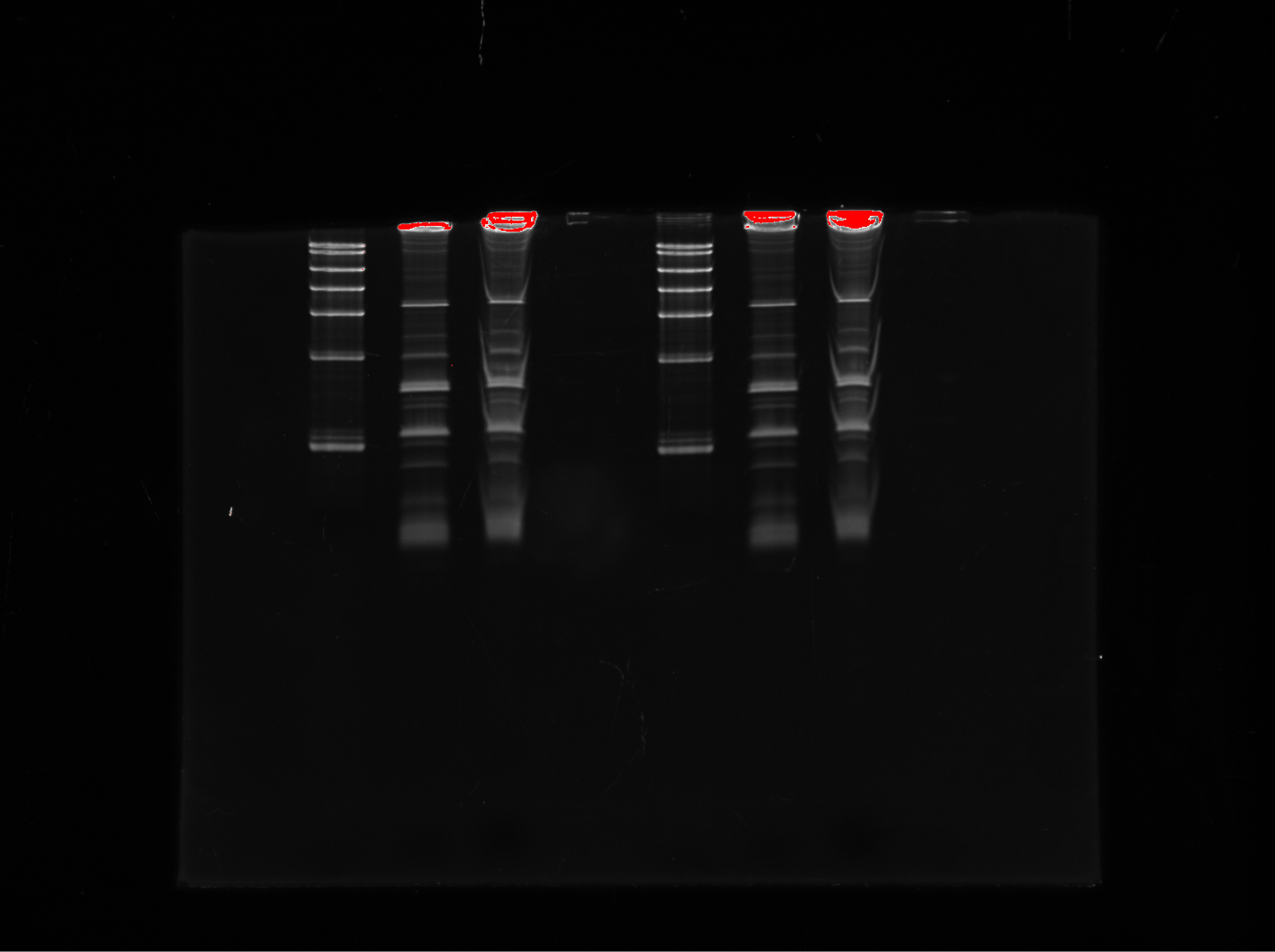

Supplement: Figure 6—source data 2. [file elife-106846-fig6-data2.zip › 2024-0821-091801 (EtBr staining).tif]

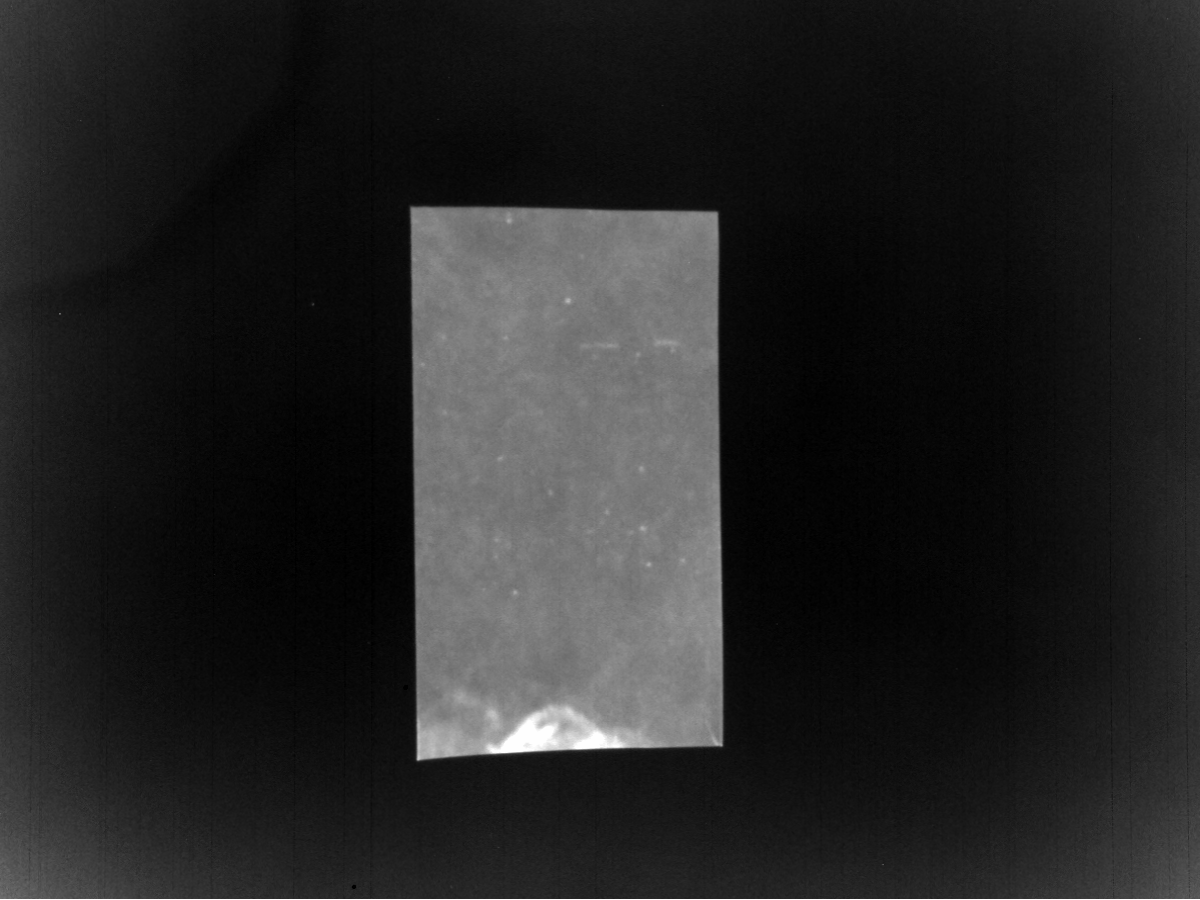

Supplement: Figure 6—source data 2. [file elife-106846-fig6-data2.zip › 2024-0821-091801 (SNORA13).tif]

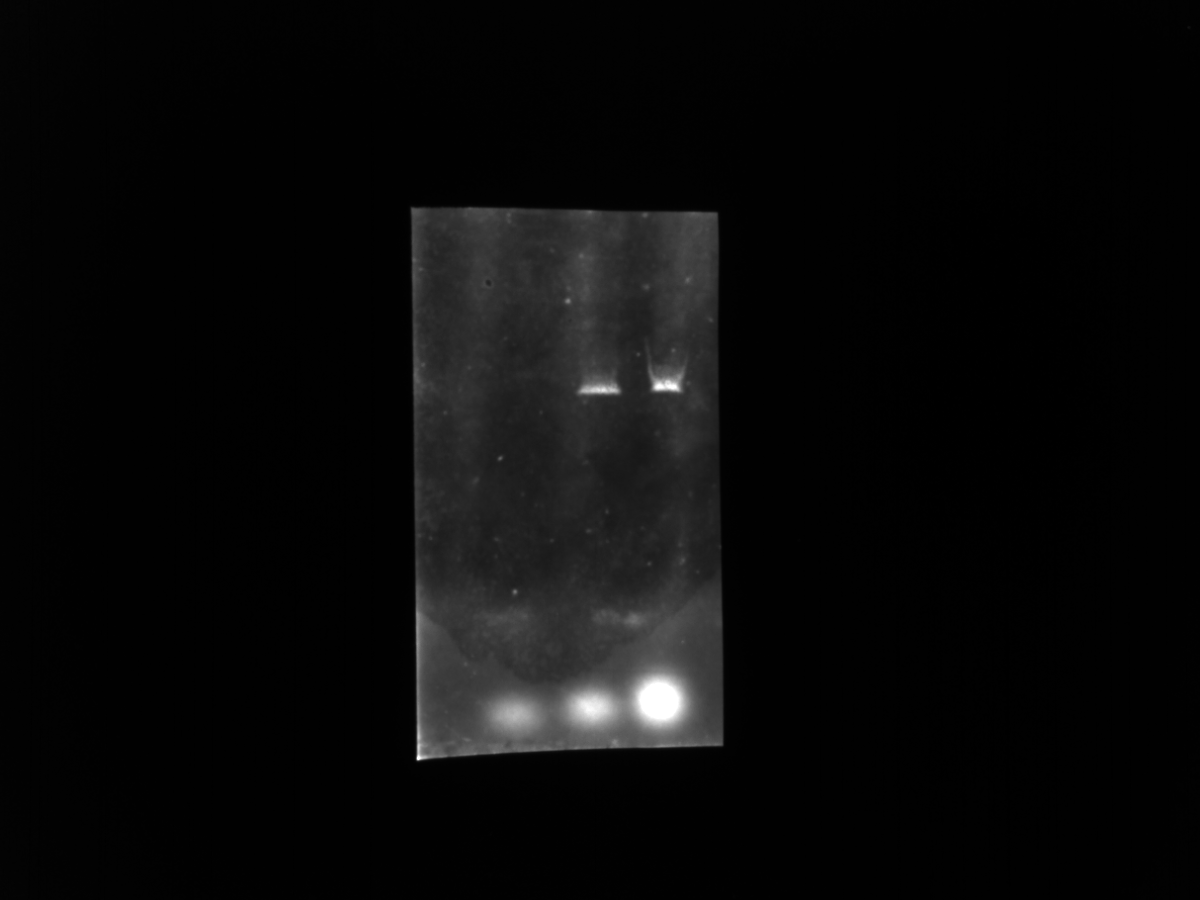

Supplement: Figure 6—source data 2. [file elife-106846-fig6-data2.zip › 2024-0821-091802 (U6).tif]

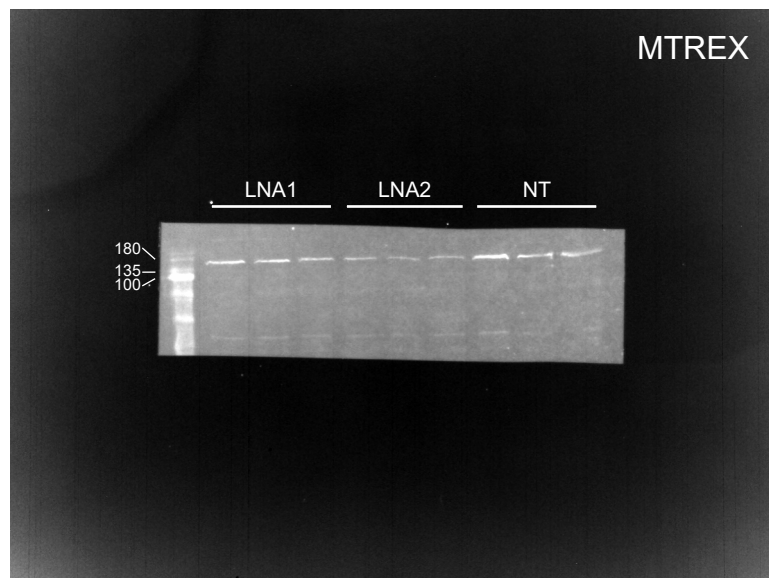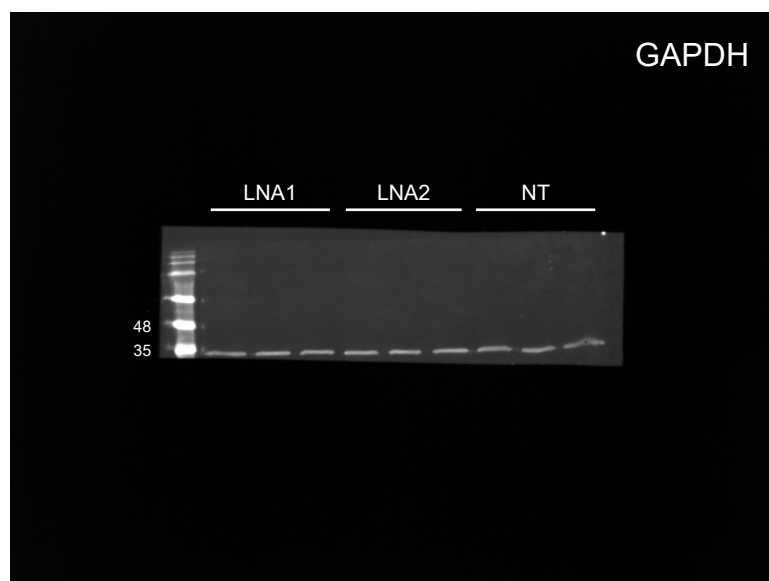

Supplement: Figure 9—figure supplement 1—source data 1. [file elife-106846-fig9-figsupp1-data1.zip › Figure 9-figure supplement 1-source data 1.pdf]
